# Editorial: Transcriptional regulation of macrophage function

**DOI:** 10.3389/fimmu.2023.1321064

**Published:** 2023-11-02

**Authors:** Zsolt Czimmerer, Andreas Patsalos, Marten A. Hoeksema

**Affiliations:** ^1^ Institute of Genetics, HUN-REN Biological Research Centre, Szeged, Hungary; ^2^ Department of Medicine, Johns Hopkins University School of Medicine, Institute for Fundamental Biomedical Research, Johns Hopkins All Children’s Hospital, St. Petersburg, FL, United States; ^3^ Department of Biological Chemistry, Johns Hopkins University School of Medicine, Institute for Fundamental Biomedical Research, Johns Hopkins All Children’s Hospital, St. Petersburg, FL, United States; ^4^ Department of Medical Biochemistry, Amsterdam Immunity and Infection: Inflammatory Diseases, Amsterdam Cardiovascular Sciences: Atherosclerosis & Ischemic Syndrome, Amsterdam UMC location University of Amsterdam, Amsterdam, Netherlands

**Keywords:** transcriptional regulation, macrophages, transcription factors, macrophage ontogeny, macrophage responses

## Macrophage origin, phenotypes, and functions

Macrophages are pivotal players in regulating immunity and maintaining tissue homeostasis. Dysregulation of their activities can contribute to a broad spectrum of human diseases. The phenotype and function of tissue-resident macrophages are intricately shaped by both their origin and local environment (1, 2).

Traditionally believed to originate solely from hematopoietic stem cells, research has revealed that certain macrophage populations arise from the yolk sac (3). In specific tissues, these embryonically derived macrophages possess the remarkable ability to self-renew, while in others, they are periodically replaced by circulating monocytes. During disease states, inflammatory monocytes can be recruited in larger numbers and subsequently differentiate into macrophages (4).

Beyond their origins, macrophages exhibit distinct phenotypic characteristics influenced by the microenvironment in which they reside. Local tissue cues play a pivotal role in determining macrophage function and phenotype, primarily through the regulation of gene transcription (5). As an illustrative example, in the peritoneal cavity, retinoic acid serves as a critical driver activating the tissue-specific peritoneal macrophage gene program through the transcription factor GATA6 (6).

In addition to tissue-specific factors, signals emanating from pathological states profoundly impact macrophage phenotypes. These signals encompass a wide array of sterile factors, including cytokines and growth factors, and non-sterile Pathogen-Associated Molecular Patterns (PAMPs) like bacterial lipopolysaccharides (LPS), viral double-stranded RNA, fungal cell wall components (e.g., beta-glucans), and bacterial flagellin. Furthermore, macrophages are frequently exposed to Damage-Associated Molecular Patterns (DAMPs), typically released by damaged or stressed tissues/cells. Such stimuli play a pivotal role in modulating macrophage phenotypes during disease progression (6).

## Transcriptional regulation

The orchestration of gene expression in macrophages is a finely tuned process governed by an intricate network of many different actors. The key to the macrophage subtype specification is the lineage-determining and stimulus-activated transcription factors. Together with collaborative transcription factors and other transcriptional regulators like epigenetic enzymes, chromatin remodeling factors, co-activators, or co-repressors, they activate enhancers and promoters. They thereby determine the transcriptional output and regulate cellular responses and the phenotype of the macrophage in response to various triggers and stimuli.

In this Research Topic, we would like to further our knowledge of transcriptional mechanisms that can regulate macrophage function *in vitro* and *in vivo*, in health and disease. Below, we have highlighted some of the main findings of the articles published in this Research Topic:

- Yashchenko et al. identified four major subsets of monocytes and two major subsets of kidney resident macrophages (KRM) using single-cell RNA sequencing. One sub-population of KRMs displayed high *Ccr2* expression, suggesting monocyte origin. Using fate mapping, they showed that less than 50% of *Ccr2^+^* KRMs are derived from Ly6C^hi^ monocytes. Interestingly, *Ccr2^+^* KRMs are almost exclusively found in the kidney cortex. They found that CX3CR1 regulated the cortex-specific accumulation of *Ccr2* ^+^ KRMs and that loss of *Cx3cr1* reduced cystic kidney disease.
- Nagenborg et al. studied the transcription factor STAT5A in macrophages. STAT5 is activated by the growth factor GM-CSF, and the authors show that targeting STAT5A blunted the transcription of genes involved in immune responses. These findings were confirmed in ex vivo tissue slices of advanced human atherosclerotic plaques as TNF, IL-8, and IL-10 secretion were affected by STAT5A inhibition. Altogether, their data showed that STAT5A is an important determinant of macrophage inflammatory responses that may be targeted to diminish atherosclerotic plaque inflammation.
- Hillman et al. characterized the monocytes derived from blood samples of active tuberculosis patients at diagnosis and mid-treatment and healthy controls by single-cell transcriptomics and functional assays. The authors show the increased frequency of CD14^+^CD16^-^ and intermediate CD14^+^CD16^+^ monocytes in ATB patients at diagnosis with upregulation of interferon signaling gene signature and many changes in the transcriptionally heterogeneous intermediate CD14^+^CD16^+^ monocytes, including upregulated MHC-II and inflammatory gene expression, and T cell activating capacity. These monocyte characteristics have a transient nature. Overall, they identified novel monocyte subsets with specific gene signatures and functional features associated with active tuberculosis.
- Sampath et al. determined the miRNA expression profile of monocytes derived from the tuberculosis disease spectrum, including drug-resistant, drug-sensitive, and latent tuberculosis patients and healthy individuals. The identified disease state-specific miRNA expression signature provides valuable information about different pathological aspects of tuberculosis, such as altered monocyte function, drug resistance, and disease severity.
- Avila-Ponce de León et al. developed and tested a mathematical model to understand how pro- and anti-inflammatory imbalance emerges in cancer. This model allows us to investigate macrophage differentiation and plasticity in the complex and continuously changing molecular microenvironment, considering different concentrations of cytokines and transcription factors.
- Domokos et al. studied the epigenetic and transcriptional bases of the angiogenesis-modulating program in alternatively polarized macrophages. The authors found that the alternative macrophage polarizing signal IL-4 simultaneously inhibits the pro-angiogenic *Vegfa* and induces the antiangiogenic *Flt1* expression in murine bone marrow-derived macrophages in STAT6 and EGR2 transcription factor-dependent manner under normoxic and hypoxic conditions. This phenomenon is also observed in distinct murine tissue-resident macrophage populations and *in vivo* parasite infection models with minor differences and is partially evolutionarily conserved in humans.
- Novita et al. characterized the expression pattern of CCR2, CCL5, IL-6, IL-10, STAT3, and SOCS3 in macrophages from tuberculous lymphadenitis patients, as there was no data on the expression of those markers. The characterization of inflammatory markers is critical to understanding the pathogenesis of tuberculous lymphadenitis. They found a high expression of CCR2, CCL5, and IL-6, while IL-10, STAT3, and SOCS3 were expressed lowly. Further investigations are needed to elucidate the detailed immunological mechanism of tuberculous lymphadenitis.
- Although previously studied in T cells and DCs, Cao et al. are the first to study Ocilrp2 in macrophages. *Ocilrp2* knockdown resulted in the increased expression of IL-6 in LPS-stimulated peritoneal macrophages. They found that Ocilrp2-related Syk activation is responsible for expressing inflammatory cytokines in LPS-stimulated macrophages. Altogether, they found that Ocilrp2 has a critical role in inflammatory signaling and identified crosstalk between Toll-like receptor and Syk signaling.
- Wierenga et al. studied how the omega-3 PUFA docosahexaenoic acid (DHA) influences TLR4-driven pro-inflammatory and IFN1-regulated gene expression in a novel self-renewing murine fetal liver-derived macrophage (FLM) model. They found that DHA modestly downregulated LPS-induced expression of NF-κB-target genes. Importantly, DHA resulted in the loss of a subset of FLMs that highly expressed NF-κB- and IRF7/STAT1/STAT2-target genes. Their data indicates that DHA potently targets both the NF-κB and interferon responses.
- Cui et al. found that providing excess iron as soluble ferric ammonium citrate (FAC) rather than as heme-iron complexes derived from stressed red blood cells (sRBC) interferes with macrophage differentiation and phagocytosis. Their findings suggest that high levels of non-heme iron interfere with macrophage differentiation by inducing mitochondrial oxidative stress. This may be relevant in diseases like chronic obstructive pulmonary disease (COPD), where both iron overload and defective macrophage function have been suggested to play a role in pathogenesis.
- Cai et al. investigate whether high iodine can cause macrophage polarization imbalance. High iodine can increase *HK3* expression in macrophages and promote macrophage polarization towards a pro-inflammatory (M1-like) phenotype. Targeting HK3 was shown to inhibit this pro-inflammatory phenotype.
- Mauduit et al. analyzed chronic inflammation in a mouse model of Sjögren’s syndrome (SS). Their thorough analysis of bulk RNA-seq and spatial gene expression suggests that altered metabolism and the hallmarks of inflammatory responses from both epithelial and immune cells drive inflammation.
- Macrophage polarization towards tumor-associated macrophages is activated by stimuli from the tumor microenvironment that are relayed to the nucleus through membrane receptors and signaling pathways that result in gene expression reprogramming in macrophages. In their review, Kerneur et al. focus on the main signaling pathways involved in macrophage polarization activated upon ligand-receptor recognition and in the presence of other immunomodulatory molecules in cancer.
- Nott and Holtman discuss key concepts and challenges in the post-genome-wide association studies (GWAS) interpretation of Alzheimer’s disease (AD) and Parkinson’s disease (PD) GWAS risk alleles, with a particular focus on microglia. Microglia, the tissue-resident macrophages of the brain, are essential for brain health and have links to various brain disorders. Post-GWAS research aims to understand how genetic risk factors impact microglia function and disease susceptibility. Challenges include identifying target cell types, causal variants, and relevant genes. Many risk GWAS risk alleles can affect microglia. Understanding their role in microglia function is vital for a deeper comprehension of these disorders.
- Koncz et al. discuss the role of macrophages in sterile inflammation triggered by DAMPs, which in turn leads to macrophage activation. This process is of interest as a better understanding of the contribution of macrophage subtypes to sterile inflammation can offer insights into manipulating their phenotypic transition from inflammation to resolution in sterile inflammatory diseases.

We hope these findings will lead to a better understanding of how the macrophage’s phenotype is regulated and can lead to the identification of novel regulators of (disease- or signal-activated) gene transcription programs in macrophages. In the future, this may result in novel intervention strategies targeting macrophage transcriptional programs in disease.

## Author contributions

ZC: Writing – original draft, Writing – review & editing. AP: Writing – review & editing. MH: Conceptualization, Writing – original draft, Writing – review & editing.
